# Strong Labour Market Inequality of Opportunities at the Workplace for Supporting a Long and Healthy Work-Life: The SeniorWorkingLife Study

**DOI:** 10.3390/ijerph16183264

**Published:** 2019-09-05

**Authors:** Lars L. Andersen, Per H. Jensen, Annette Meng, Emil Sundstrup

**Affiliations:** 1 National Research Centre for the Working Environment, DK-2100 Copenhagen, Denmark (A.M.) (E.S.); 2 Centre for Comparative Welfare Studies, Aalborg University, DK-9220 Aalborg, Denmark

**Keywords:** senior worker, aging, occupational health, public health, workplace, sustainable employment

## Abstract

Most European countries are gradually increasing the state pension age, but this may run counter to the capabilities and wishes of older workers. The objective of this study is to identify opportunities in the workplace for supporting a prolonged working life in different groups in the labour market. A representative sample of 11,200 employed workers ≥ 50 years responded to 15 questions in random order about opportunities at their workplace for supporting a prolonged working life. Respondents were stratified based on the Danish version of the International Standard Classification of Occupations (ISCO). Using frequency and logistic regression procedures combined with model-assisted weights based on national registers, results showed that the most common opportunities at the workplace were possibilities for more vacation, reduction of working hours, flexible working hours, access to treatment, further education and physical exercise. However, ISCO groups 5–9 (mainly physical work and shorter education) had in general poorer access to these opportunities than ISCO groups 1–4 (mainly seated work and longer education). Women had poorer access than men, and workers with reduced work ability had poorer access than those with full work ability. Thus, in contrast with actual needs, opportunities at the workplace were lower in occupations characterized by physical work and shorter education, among women and among workers with reduced work ability. This inequality poses a threat to prolonging working life in vulnerable groups in the labour market.

## 1. Introduction

Since the 1980s, the combination of increasing lifespan and decreasing birth rates have led to marked demographic changes in most European countries with a larger proportion of older adults above the official retirement age [1,2]. As a consequence, politicians in most Western countries have implemented reforms to increase the retirement age in an attempt to balance the increased economic costs of an ageing population [3,4]. The most far-reaching pension reform in Europe has occurred in Denmark, where the state pension age has been set to life expectancy minus 14.5 years, meaning that state pension age is expected to increase gradually from 65 years in 2018 to 67 years in 2022 and further to 74.5 years in 2070 [5]. Such a drastic reform can be considered a large-scale natural experiment involving millions of people and should be closely followed and evaluated.

While the Danish pension reform makes sense from an economic point of view, it should be investigated whether people will be able or willing to work until they reach 74.5 years of age. Calculations conducted by the European Commission [6] estimate that in 2070 the average age of retirement will be 68, indicating that there will be a large gap between the expected average age of retirement and the state pension age. This gap occurs due to push [7] and jump [8] factors. Push refers to involuntary retirement, e.g., due to poor health. Studies indicate that the number of years lived in good health is lagging behind the increase in life expectancy [9], most probably because chronic health problems increase with age [10], while individual physical capacity inherently declines with age [11]. Thus, by increasing the official retirement age, more workers are likely to be pushed out of the labour market. Jump refers to some older workers developing a psychological distance from their work and identifying with non-work roles, i.e., they may find it fulfilling and more important to spend their time with their partner or to be with their grandchildren [12,13] or simply enjoy leisure time, travelling, etc. [8]. 

In Denmark, a major challenge exists in narrowing down the discrepancy between the future state pension age and the average age of retirement. This may be achieved by improving health and work ability among those subject to push factors and improving the motivation to work longer among those subject to jump. In this endeavour, the workplace may hold or create a number of opportunities for promoting health and work ability and motivating employees to stay longer. Most studies analysing workplace strategies and practices in relation to older workers use company surveys among employers — instead of employees — as the empirical basis [14,15]. However, the employer may see things in a more positive view than what is experienced by the employees. In this study, we analyse how different occupational segments of older employees perceive programmes and opportunity structures offered at the workplaces. 

Previous studies have found that there is a social gradient in health; less educated people, often with physically strenuous work, have, on average, a shorter lifespan, fewer years in good health and reduced work ability as compared to higher educated segments of the workforce [16,17,18,19,20]. Especially women with short or no education and high physical work demands are vulnerable to be pushed out of the labour market due to poor health [21]. It is therefore of interest to know whether these differences correspond to differences in programmes and opportunity structures offered at the workplaces. That is, are opportunities at the workplace to prolong working life more prevalent among occupational groups dominated by high as compared to low educated segments of the labour force, or is it more prevalent among older workers with good work ability as compared to older workers with poorer work ability? And what is the role of gender? These research questions have so far been underappreciated. 

Therefore, the aim of this paper is to assess opportunities in the workplace for supporting a prolonged working life in different groups of the labour market. The analyses use representative data from Danish workers aged +50 years participating in the SeniorWorkingLife study. 

## 2. Materials and Methods 

### 2.1. Study Design

The SeniorWorkingLife study is registered as a cohort study in ClinicalTrials.gov (Identifier: NCT03634410) and the first wave was carried out between July and October 2018 [22]. For the first wave, Statistics Denmark drew a probability sample of 30,000 Danes ≥ 50 years (18,000 employed, 7000 unemployed, 3000 on voluntary early retirement, 2000 on disability pension). Potential participants received an invitation and a personal questionnaire-link via an online digital mailbox linked to their Danish social security number (e-Boks). Subsequently, we merged the survey responses with high-quality national registers about occupational groups (Danish version of ISCO) through the unique social security number assigned to all Danish residents at birth or immigration. For the analyses of the present paper, we only included currently employed wage earners. Among these, the response percentage to the full-length questionnaire was 56% (i.e., those also responding to other questions about, e.g., work environment and health not included in the present article). However, those who replied only partly (i.e., to the questions mentioned below) were also included in the present analyses. Further, only those registered in ISCO groups 1 to 9 were included, i.e., excluding ISCO group 0 (Armed Forces Occupations) due to a small number of observations (*n* = 56). Thus, the total sample size included in the present analyses was 11,200 (~62% of 18,000) currently employed workers of ISCO groups 1 to 9 responding to the questions about available opportunities at their workplace. Table 1 shows characteristics of the included population.

### 2.2. Stratification into Occupational Groups

We stratified respondents into nine occupational groups based on the official Danish version of the International Standard Classification of Occupations (ISCO) [23]. The Danish ISCO is a six-digit classification, structured as a five-level hierarchical structure based on information from high-quality national registers at Statistics Denmark, and divides the Danish labour market into 563 professional groups, each containing a number of closely related work functions. The skill requirements in each ISCO group range from I (most basic) to IV (most advanced). For the present study, we used the first-level ISCO groups: (1) Managers (level III and IV skill requirement), (2) Professionals (level IV skill requirement), (3) Technicians and Associate Professionals (level III skill requirement), (4) Clerical Support Workers (level II skill requirement), (5) Services and Sales Workers (level II skill requirement), (6) Skilled Agricultural, Forestry and Fishery Workers (level II skill requirement), (7) Craft and Related Trades Workers (level II skill requirement), (8) Plant and Machine Operators and Assemblers (level II skill requirement) and 9) Elementary Occupations (level I skill requirement). The majority of ISCO groups 1–4 have seated work (76%, 57%, 74% and 75%, respectively), and the majority of ISCO groups 5–9 have physical work (86%, 83%, 89%, 72% and 89%, respectively). 

### 2.3. Questionnaire

Participants responded to multiple-choice questions about opportunities at the workplace, with fifteen response options provided in random order, e.g., senior counselling, reduction of working hours, flexible working hours, development of competencies, additional days off, reduced workload and responsibility, better salary and health promotion offers [22]. The option ‘none of the above’ was given at the bottom of the multiple-choice questions as the sixteenth option. All the response options are shown in Table 2. 

Furthermore, the participants were asked to rate their work ability compared with lifetime best; *“Please rate your current work ability on a scale of 0–10, where 0 is unable to work and 10 is lifetime best work ability”*. The concept of work ability was developed by Ilmarinen to reflect the balance between human resources and the demands of work. This question has previously been validated in relation to a number of health outcomes, e.g., the risk of future disability pension [24]. Good work ability was defined as a score of 8–10 on the 0–10 scale [24].

### 2.4. Statistics

Using the SurveyFreq procedure of SAS version 9.4 (SAS institute, Cary, North Carolina, USA), we produced estimates of prevalence (percentage) and 95% confidence intervals, and using the SurveyLogistic procedure we produced estimates of odds ratios (OR) and 95% confidence intervals for the chance of choosing each different option of the multiple-choice questionnaire (i.e., having the opportunity or not). Thus, we used a binary logit model (Optimization technique: Fisher’s scoring. Variance adjustment: degrees of freedom). In contrast to the ‘normal’ frequency and logistic procedures of SAS, the SurveyFreq and SurveyLogistic procedures take into account sampling clusters and strata. Analyses were controlled for age, sex and ISCO group. For sex, men were used as a reference, i.e., ORs for women. For occupation, ISCO group 1–4 (mainly seated work) was used as a reference, i.e., estimates represent ORs for group 5–9 (mainly physical work). For work ability, individuals with good work ability (8–10 on a scale of 0–10) were used a reference, i.e., estimates represent ORs for individuals with at least 30% reduced work ability (0–7 on a scale of 0–10). Model-assisted weights were used for the SurveyFreq and SurveyLogistic procedures to produce representative estimates and 95% confidence intervals. These weights were based on information from high-quality national registers (Statistics Denmark) and accounted for age, sex, highest completed education, occupational industry, family type, family income and origin [22]. 

## 3. Results

Table 1 shows the descriptive background information of the population divided by ISCO groups 1–4 and 5–9 and by sex. There were both similarities and differences between the groups. For example, age, lifestyle and weekly work hours were quite similar across the groups, whereas work-related physical activity and the proportion of workers with reduced work ability were much higher among ISCO groups 5–9 than groups 1–4. 

**Figure 1:**
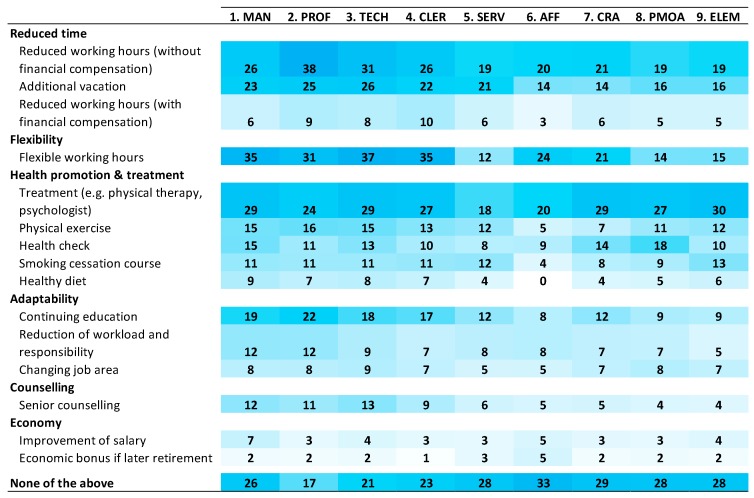
Colour-intensity map of available opportunities at the workplace in the 9 ISCO groups. Prevalence (percentage of respondents) is provided in each box. 1. Managers (MAN), 2. Professionals (PROF), 3. Technicians and Associate Professionals (TECH), 4. Clerical Support Workers (CLER), 5. Services and Sales Workers (SERV), 6. Skilled Agricultural, Forestry and Fishery Workers (AFF), 7. Craft and Related Trades Workers (CRA), 8. Plant and Machine Operators and Assemblers (PMOA), 9. Elementary Occupations (ELEM).

Figure 1 shows a colour-intensity map of opportunities at the workplace in the nine separate ISCO groups. The most common opportunities were more vacation, reduction of working hours, flexible work hours, access to treatments (e.g., physical therapy, psychologist), further education and physical exercise. However, the overall picture based on the colour-intensity map shows that ISCO groups 5–9 (mainly physical work and shorter education) in general, have poorer access to these than ISCO groups 1–4 (mainly seated work and longer education). 

Table 2 and Table 3, respectively, show the prevalence (percentage) and odds ratios (ORs) of opportunities at the workplace. Table 2 shows that the most widespread opportunities were reduced time, flexible working hours, additional vacation and health promotion; however, the ORs calculations of Table 3 show that these were not equally distributed across groups. Thus, Table 3 shows that ISCO groups 5–9 compared with 1–4 were less likely to have access to 11 out of the 15 different opportunities (ORs ranging from 0.33 to 0.87), and were more likely not to have any of the opportunities at all (OR 1.57). Women compared with men were less likely to have access to 11 out of the 15 opportunities (ORs ranging from 0.51 to 0.83). Individuals with reduced work ability compared with good work ability were less likely to have access to 9 out of the 15 opportunities (ORs ranging from 0.63 to 0.81), and were more likely not to have any of the opportunities at all (OR 1.21). Individuals with reduced work ability were more likely to only have access to 1 out of the 15 opportunities (reduced working hours with financial compensation, OR 1.27).

## 4. Discussion

The main finding of this study is that a clear Matthew effect exists, i.e., those with the greatest needs have the poorest possibilities at the workplace for supporting a long and healthy work-life. That is, those at highest risk of being pushed out of the labour market, i.e., workers in job groups characterized by short education and physical work, as well as those with reduced work ability, had fewer opportunities in general. Opportunities at the workplace targeting mainly the best-off seniors may reduce the numbers of employees being pushed out of the labour market only to a very limited extent, and the challenge of raising the retirement age remains unresolved.

The most widespread opportunities reported were reduced time, flexible working hours, additional vacation and health promotion, but these were not equally distributed across the different segments. Reduced working hours can be one way to reduce the overall workload and thereby reduce the risk of push in workers with high work demands and poor health, which can be especially relevant for many employees in ISCO groups 5–9. However, only about 19–20% of those in ISCO groups 5–9 (Table 2) had the opportunity for reduced working hours and even fewer (5–6%) with financial compensation. It should be investigated if a more flexible system with a gradual reduction in working hours with financial compensation could be a cost-beneficial method of prolonging working life, especially among those with high physical work demands. Reduced working hours, more vacation and flexible working hours may also be a way to reduce jump, as more leisure time and freedom to choose may reduce the desire to completely leave the labour market for those in good health, which is the case for many older workers in ISCO groups 1–4. 

Opportunities for workplace health promotion and treatment possibilities were also poorer in ISCO groups 5–9 than in groups 1–4. Of the specific workplace health promotion offers, physical exercise was possible only for 10–17% across all ISCO groups (Table 2). This is surprising, as there is consistent evidence through systematic reviews of positive effects of physical exercise for health [25], also when performed at the workplace [26,27,28]. Thus, a challenge still exists in communicating this knowledge to workplaces, both those with seated and physical work. National campaigns targeted at workplaces may be a way forward [29]. Health technology may also be a strategy to promote increased physical activity at the workplace among those with seated work [30]. 

In ISCO groups 1–4 and 5–9, respectively, 23% and 38–40% of the older workers had reduced work ability compared to their lifetime best. Prospective cohort studies with register follow-up show that reduced work ability is a major push factor, markedly increasing the risk of involuntary early retirement [24,31]. Thus, opportunities for health promotion and better working conditions are especially important for employees with reduced work ability. The present study shows that the reality in workplaces is the exact opposite, i.e., for 9 out of 15 possibilities (Table 3) employees with reduced work ability had fewer opportunities (ORs ranging from 0.63 to 0.81) in the workplace to support a prolonged and healthy work-life, i.e., lower access to possibilities such as physical exercise, health checks, healthy diet, flexible working hours and changing job area. Previous studies show that lack of physical exercise, poor musculoskeletal capacity and obesity, as well as high mental and physical work demands, and lack of autonomy increase the risk for poor work ability [32]. Although causal inferences should be drawn with care, lack of opportunities at the workplace may be part of the reason for the reduced work ability in these employees. Regardless of the direction of causality, providing relevant opportunities at the workplace may be used to assist workers with reduced work ability, and thereby prevent them from being pushed out of the labour market. However, in some cases it may be easier for workplaces to replace than support workers with reduced work ability, especially in job groups characterized by short education. Of all the available opportunities, workers with reduced work ability only had better possibilities for reduced working hours with financial compensation, likely because, in Denmark, compensation for reduced working hours in certain types of jobs (e.g., flex jobs and sheltered jobs) is paid by the municipalities and not by the employer.

Another interesting finding is that men and women do not seem to have equal opportunities. Thus, for 11 out of 15 possibilities (Table 3) women were offered measures to a lesser extent than men (ORs ranging from 0.51 to 0.86) that may help them prolonging working life. Such differences can be the outcome of a strong horizontal gender division of labour in the Danish labour market, meaning that men and women are positioned in different occupations and subject to different industrial relations regulations. It should also be mentioned that due to jump reasons, women retire earlier than men [8]. Therefore, if the overall aim is to increase the employment rate among older workers it is important that employers develop measures that meet the needs of women, e.g., measures to improve work–life balance and in general provide more opportunities at the workplace for prolonging working life. At the same time, women with short education and high physical work demands are more likely to be pushed out due to poor health [21]. This highlights the need for effective workplace policies to ensure a prolonged working life especially for this group of workers.

### Strengths and Limitations

This study has both strengths and limitations. Non-response is always a limitation when attempting to produce representative estimates in this type of study. However, to ensure representative estimates, Statistics Denmark drew a probability sample among all eligible Danish residents age 50 years or older and combined this with model-assisted weights based on high-quality national registers. Thus, we can be fairly certain that the estimates are representative of workers in Denmark aged 50 years or older. The ISCO system is used internationally to group occupations containing a number of closely related work functions and is a better predictor of health outcomes than socioeconomic class [23]. The Danish version of ISCO is based on high-quality registers from Statistics Denmark and is highly reliable. Thus, using ISCO to classify occupations is a strength of the study. Additionally, a lack of knowledge of existing opportunities at the workplace may have also influenced the responses in the present study, highlighting the potential of communicating company policies, and opportunities to the workers. Finally, a follow-up in national registers on labour market attachment in the years to come is necessary to investigate whether the factors included in the present study can actually help to prolong working life.

## 5. Conclusions

In contrast with actual needs, workplace opportunities for supporting a prolonged work life were lower among older workers in occupations with physical work, in women and in workers with reduced work ability. This inequality poses a threat to prolonging working life in vulnerable groups in the labour market.

## Figures and Tables

**Table 1:** Demographics, lifestyle, work and work ability among men and women in ISCO group 1–4 (mainly seated work) and 5–9 (mainly physical work), respectively. Results are either mean (SD) or prevalence as a percentage (95% CI).

Variable	ISCO 1–4 Mainly Seated Work		ISCO 5–9 Mainly Physical Work	
	Men	Women	Men	Women
*N*	3418	3823	2543	1416
Age (mean)	56.9 (5.8)	56.2 (4.9)	56.9 (5.5)	56.2 (4.5)
Smoking (% yes)	14 (13–15)	14 (13–16)	26 (24–28)	26 (24–29)
Alcohol (% men > 14 and % women > 7 per week)	16 (15–17)	18 (17–19)	12 (10–13)	9 (8–11)
BMI (mean)	26.6 (4.3)	25.4 (5.0)	27.4 (4.8)	26.0 (5.8)
Physical activity work (%)				
1. Seated	70 (68–71)	63 (62–65)	19 (17–21)	10 (8–12)
2. Standing or walking	21 (20–23)	26 (24–28)	24 (22–26)	23 (20–25)
3. Standing or walking with a lot of lifting or carrying	8 (7–9)	9 (8–10)	43 (40–45)	52 (49–55)
4. Heavy or fast work that is physically strenuous	1 (1–2)	1 (1–2)	14 (12–16)	15 (13–17)
Weekly working hours (mean)	41.0 (9.1)	37.3 (7.6)	39.1 (10.1)	35.0 (7.4)
Expected retirement age (mean)	67.5 (5.0)	66.2 (3.5)	66.8 (4.4)	65.7 (3.0)
Reduced work ability (%)	23 (21–24)	23 (21–24)	40 (38–42)	38 (35–41)

**Table 2:** Availability of different opportunities at the workplace among men and women in ISCO groups 1–4 and 5–9, respectively, provided as prevalence (percentage of respondents) and 95% confidence intervals. ORs for ISCO group 5–9 (ref: ISCO groups 1–4) and women (ref: men) are provided in the last two columns.

Available Opportunities at the Workplace	ISCO 1–4 Mainly Seated Work		ISCO 5–9 Mainly Physical Work	
	Men	Women	Men	Women
	*n = 3418*	*n = 3823*	*n = 2543*	*n = 1416*
**Reduced time**				
Reduced working hours (without financial compensation)	34 (32–35)	32 (30–33)	20 (18–22)	19 (17–21)
Additional vacation	25 (24–27)	24 (22–25)	17 (15–18)	18 (16–21)
Reduced working hours (with financial compensation)	9 (8–10)	8 (7–9)	6 (5–7)	5 (3–6)
**Flexibility**				
Flexible working hours	37 (35–39)	30 (28–32)	17 (16–19)	11 (9–13)
**Health promotion & treatment**				
Treatment (e.g., physical therapy, psychologist)	30 (28–32)	23 (22–25)	26 (24–28)	22 (19–24)
Physical exercise	17 (16–19)	13 (12–15)	10 (8–11)	12 (10–14)
Health check	15 (14–17)	8 (7–9)	14 (12–16)	8 (7–10)
Smoking cessation course	11 (10–12)	11 (10–12)	10 (9–12)	12 (10–14)
Healthy diet	10 (9–11)	6 (5–7)	5 (4–6)	4 (3–5)
**Adaptability**				
Continuing education	20 (19–22)	20 (18–21)	10 (9–12)	12 (10–14)
Reduction of workload and responsibility	13 (12–14)	8 (7–9)	7 (6–8)	6 (5–8)
Changing job area	10 (9–11)	6 (5–7)	8 (6–9)	5 (4–7)
**Counselling**				
Senior counselling	14 (13–15)	9 (8–10)	5 (4–6)	4 (3–5)
**Economy**				
Improvement of salary	4 (4–5)	3 (3–4)	4 (3–4)	3 (2–4)
Economic bonus if later retirement	2 (2–3)	1 (1–2)	3 (2–3)	2 (1–3)
**None of the above**	19 (17–20)	21 (20–23)	28 (26–31)	28 (25–30)

**Table 3:** Odds ratios and 95% confidence intervals for the availability of different opportunities at the workplace in ISCO 5–9 vs. 1–4, among women vs. men, and among those with reduced work ability vs. those with full work ability. Values less than 1 mean that there is less of a chance of having that opportunity at the workplace. Statistically significant findings are marked in bold.

Available Opportunities at the Workplace	OR (95% CI) ^a^		
	ISCO 5–9 vs. 1–4	Women vs. Men	Reduced vs. Full Work Ability
**Reduced time**			
Reduced working hours (without financial compensation)	**0.50 (0.45–0.55)**	0.94 (0.85–1.03)	0.98 (0.88–1.09)
Additional vacation	**0.64 (0.57–0.72)**	0.96 (0.87–1.07)	**0.81 (0.72–0.91)**
Reduced working hours (with financial compensation)	**0.62 (0.51–0.74)**	**0.83 (0.71–0.97)**	**1.27 (1.06–1.51)**
**Flexibility**			
Flexible working hours	**0.33 (0.30–0.37)**	**0.69 (0.63–0.76)**	**0.74 (0.66–0.84)**
**Health promotion & treatment**			
Treatment (e.g. physical therapy, psychologist)	**0.87 (0.79–0.97)**	**0.72 (0.65–0.79)**	0.92 (0.83–1.03)
Physical exercise	**0.65 (0.57–0.75)**	**0.86 (0.76–0.97)**	**0.77 (0.66–0.89)**
Health check	0.92 (0.80–1.06)	**0.51 (0.44–0.58)**	**0.81 (0.69–0.94)**
Smoking cessation course	1.00 (0.86–1.15)	1.06 (0.93–1.22)	0.89 (0.76–1.04)
Healthy diet	**0.54 (0.44–0.66)**	**0.63 (0.53–0.75)**	**0.71 (0.58–0.88)**
**Adaptability**			
Continuing education	**0.48 (0.42–0.55)**	0.98 (0.88–1.10)	**0.78 (0.68–0.90)**
Reduction of workload and responsibility	**0.60 (0.51–0.71)**	**0.65 (0.56–0.75)**	0.99 (0.83–1.17)
Changing job area	**0.75 (0.63–0.90)**	**0.58 (0.49–0.69)**	**0.63 (0.51–0.77)**
**Counselling**			
Senior counselling	**0.38 (0.31–0.45)**	**0.61 (0.53–0.71)**	**0.73 (0.61–0.88)**
**Economy**			
Improvement of salary	0.83 (0.65–1.06)	**0.71 (0.56–0.89)**	**0.70 (0.53–0.94)**
Economic bonus if later retirement	1.31 (0.96–1.80)	**0.64 (0.46–0.90)**	0.71 (0.49–1.04)
**None of the above**	**1.57 (1.42–1.75)**	1.07 (0.96–1.18)	**1.21 (1.08–1.36)**

a, adjusted for gender, age and ISCO group.

## Data Sharing Statement

Researchers interested in using the data should contact the project leader Prof. Lars L. Andersen.
